# Pediatric Safety of Polysorbates in Drug Formulations

**DOI:** 10.3390/children7010001

**Published:** 2019-12-20

**Authors:** Christina Kriegel, Matthias Festag, Ravuri S.K. Kishore, Dieter Roethlisberger, Georg Schmitt

**Affiliations:** 1 F. Hoffmann-La Roche, Ltd., 4070 Basel, Switzerland; satya_krishna_kishore.ravuri@roche.com; 2 Pharmaceutical Sciences, Roche Pharmaceutical Research and Early Development, Roche Innovation Center Basel, F. Hoffmann-La Roche, Ltd., 4070 Basel, Switzerland; matthias.festag@roche.com (M.F.);; 3 Formerly Employed by F. Hoffmann-La Roche, Ltd., 4070 Basel, Switzerland; 4 Lonza AG, Drug Product Services, 4057 Basel, Switzerland

**Keywords:** polysorbate, excipient, safety factor, pediatric, toxicology, pharmaceutical, formulation

## Abstract

Polysorbates 20 and 80 are the most frequently used excipients in biotherapeutics, the safety data for which have been well documented in adults. The polysorbate content in therapeutic formulations that are administered to children, however, has been less clearly regulated or defined with regard to safety. In pediatric patients, excessive amounts of polysorbate in biotherapeutics have been linked to hypersensitivity and other toxicity-related effects. To determine safe levels of polysorbates for young patients, we have developed the progressive pediatric safety factor (PPSF), an age- and weight-based tool that estimates the amount of parenterally administered polysorbates 20 and 80 in formulations that will avoid excipient-related adverse events. Compared with existing modalities for calculating maximum acceptable doses of excipients for initial clinical trials in pediatrics, the PPSF is far more conservative, thus constituting an added margin of safety for excipient exposure in the most sensitive subpopulations—i.e., neonates and infants. Further, the PPSF may be applied to any relevant excipient, aiding pharmaceutical developers and regulatory authorities in conservatively estimating the safety assessment of a biotherapeutic’s formulation, based on excipient levels.

## 1. Introduction

Polysorbates (PSs) are ubiquitous in biotherapeutic formulations, protecting protein drugs from interfacial stresses [1], and are generally considered to be safe within the ranges that are used in biotherapeutics [2]. Upon parenteral administration, polysorbates are known to degrade rapidly via hydrolysis due to esterases in the plasma [3]. The fatty acids are expected to further metabolize via beta-oxidation leading to formation of carbon dioxide which is exhaled. The polyoxyethylene sorbitan is expelled via urine and to a lesser degree in feces [4]. Despite the breadth of safety data on PS as an excipient in adult pharmaceutical formulations [2], little has been reported in the pediatric population, compared with the frequent exposure of children to this excipient. There is no industry-wide accepted limit on safe levels of PSs as excipients in pediatric formulations for the administration of drug products that contain PS—particularly for the parenteral route of administration to neonates and infants. Due to the immature or developing detoxification systems in these human subpopulations, understanding the limits of the use of surfactants will benefit the patient to minimize potential risks. Until now, there has been no systematic method of estimating acceptable doses of PSs for this vulnerable subpopulation.

In this report, we review existing safety data on PSs as excipients and introduce a predictive tool—the progressive pediatric safety factor (PPSF)—for approximating safe doses of PSs to pediatric patients. By applying the PPSF, clinicians, formulation experts, and regulatory authorities will be guided to derive safe and conservative levels of PSs that are tolerated by pediatric subjects, particularly with regard to formulation and medication.

## 2. Materials and Methods

The scoping review component of this article was performed through a literature search of the Pubmed database and referrals to regulatory agencies and pharmaceutical companies with regard to the prevalence, safety, and activities of polysorbates 20 and 80 and other excipients. No eligibility criteria or synthesis or analysis of data was applied.

### Pediatric Safety Factor

In the context of safety, a 10-fold adjustment factor is commonly used to allow for inter-individual human variation in response to toxic agents [5]. This factor generally covers for variability due to ethnic differences in kinetics and xenobiotic oxidation and also for age-related variability, including children [6]. Various refined methods have been proposed to derive specific adjustment factors to replace the default factor of 10 for human variability. Our approach for the use of safety assessments of PSs applies a progressive pediatric safety factor, based on age and body weight, that reflects the immaturity of major detoxification systems relevant for PSs. 

## 3. Results and Discussion

### 3.1. Potential Toxicity and Hypersensitivity in Adults Due to PS20/80: A Dosing Issue?

PS20 and PS80 are the most commonly used surfactants in biotherapeutic formulations and are also added to foods and cosmetic products as emulsifying agents. The Dailymed database refers to PS20 and PS80 as excipients in 2475 and 6326 drugs, respectively [7], with oral, parenteral, ophthalmic, and topical routes of administration. The FDA has granted PS80 the status of generally recognized as safe (GRAS), and both PSs are listed in its Inactive Ingredients Database [8]. Per the WHO, the accepted oral daily intake of PSs by adults is 25 mg/kg, compared with 10 mg/kg as suggested by the Scientific Committee on Food [9,10].

Of the currently marketed intravenous and subcutaneous drug products, amiodarone hydrochloride iv and Hectorol^®^ (doxercalciferol) iv contain the highest doses of PS80 and PS20—300 mg and 20 mg, respectively—when given without antihistamine or corticosteroid pretreatment [7]. Thus, one possible inference is that these quantities could reasonably be considered the maximum excipient doses that are proven to be well tolerated in adults—the threshold that defines their systemic toxicity. In fact, this dose of PS80 contributes to the hypotensive effects of amiodarone through its ability to induce arterial vasodilation [11].

This difference in maximum dose between PS80 and PS20 merely reflects the recent trend in the use of these excipients—not that the latter is more toxic. Over their decades-long existence, small-molecule formulations have required high doses of PS80 to solubilize or emulsify the active ingredient. The recent appearance of biological formulations, however, has shifted preferences toward PS20, primarily because its main fatty acid ester moiety is the more stable lauric acid (compared with oleic acid in PS80) [12].

Based on nonclinical data [13,14], PS20 and PS80 have been and continue to be regarded as nontoxic, nonirritant, and noncarcinogenic and are thus considered safe and well tolerated. In recent years, however, there have been reports of hypersensitivity [15] and anaphylactic shock [16] due to highly dosed PSs as excipients. Although such cases are rare, these data have renewed interest in ensuring that PS20 and PS80 be delivered to patient populations at safe and acceptable doses as excipients in biotherapeutic formulations. Today, it is recognized that PSs are associated with injection- and infusion-site adverse events, hepatotoxicity, pseudoallergy, hypersensitivity reactions, and anaphylactic shock [17,18,19,20,21,22].

The increase in hypersensitivity reactions to docetaxel is likely to have been caused by extremely high doses of PS80. Based on the FDA-approved dose of docetaxel to a prostate cancer patient, 3900 mg PS80 is coadministered on average, prompting recommendations that doses of this excipient be reduced if possible, as with the third FDA-approved taxane, cabazitaxel, which contains 957 mg PS80 per dose, per the Dailymed database when dosed according to 20 mg cabazitaxel/m^2^ body surface area of an adult (1.84 m^2^) [7,19]. The frequency and severity of such reactions are diminished when lower amounts of the allergen are injected, but hypersensitivity reactions are not limited to the administration of high doses of PS80—they can also occur with monoclonal antibodies that contain minute levels of PS80 or PS20, although it is often not clearly established whether the antibody itself or the PS is responsible.

### 3.2. Pediatric Exposure to PS80 and PS20

The safety limits that are specified for PS20 and PS80 for adults might not extrapolate easily to children. Consequently, the lack of predicted or established limits has led to issues and incidents of overdosing and adverse events in pediatric patients, beginning with the link between PS20 and PS80 and an unusual syndrome in premature neonates—characterized by thrombocytopenia, pulmonary deterioration, ascites, and liver and renal failure, secondary to vasculopathic hepatotoxicity [23]: the E-Ferol case.

Shortly after this intravenous vitamin E supplement was introduced, several clusters began observing this syndrome in a significant proportion of premature infants [24,25]; this particular product was solubilized in 9% PS80 and 1% PS20—later identified to have been extremely high levels for neonates. Notably, this syndrome developed only in newborns who received over 20 U/kg/day E-Ferol, and an association was established between these adverse events and neonates who were given PS80 and PS20 at dosages of over 72 and 8 mg/kg/day, respectively [26]. This toxicity was traced to PS80, based on its suppression of lymphocyte responses and proliferation in vitro [27] and supported by parallel nonclinical findings in dogs [28], newborn rabbits [29], and rats [30].

Since the recall of E-Ferol, there has been no such pervasive episode of PS80 toxicity, but case reports in pediatric patients have appeared periodically [31,32,33]. In Belgium, an initially healthy newborn experienced acute cardiogenic shock and multiple organ failure after receiving intravenous amiodarone at a loading dose (47 mg/kg) that was intended for oral administration—approximately 9-fold higher than the appropriate amount for intravenous infusion, underscoring the significance of properly dosing excipients for young patients [34].

Pediatric patients are potentially more sensitive to the adverse effects of PS20 and PS80 compared with the adult population. Neonates and infants are the most susceptible, because their hepatic and renal functions do not develop fully until 6–12 months and 2 years of age, respectively [35,36]; their pharmacokinetic and pharmacodynamic profiles vary substantially; and the age- and development-related pediatric safety profiles of excipients often differ from those in adults.

Due to the deficiency in safety information on excipients and the fact that many medicines are given off-label to neonates and infants, drugs that are intended for these subpopulations are often compounded from adult dosages based on body-weight or body-surface area without consideration of their immature detoxification systems. This approach, however, usually entails reformulation—and, with it, the inclusion of additional excipients—and can alter the nature of the active pharmaceutical ingredient. Some have argued for eliminating excipients from pediatric formulations altogether [37], but this option carries the risk of destabilizing the medicine, rendering this alternative impractical and nearly impossible for many drugs.

Surveys in Brazil [38], England [39], Estonia [40], and across Europe [41] have found that neonates frequently receive excipients of concern, of which PS80 is often the most commonly encountered. Although the reported frequency of PS-induced toxicity and adverse events is not substantial, there remains a patent need to supply age- and development-appropriate doses of PS for this highly sensitive group. Polypharmacy should also be considered in determining safe levels of exposure, because these young patients are commonly given more than one medication and could be exposed unwittingly to additively harmful amounts of PS.

### 3.3. Regulation of PS Use in the Pediatric Population

Until 2006, over 50% of drugs that had been administered to children had not been examined specifically for the pediatric population [42], for many reasons. Children are a heterogeneous group with regard to age and maturation status of organ systems, complicating pharmaceutical research and development. Further, the obvious ethical concerns over conducting clinical research in children have limited the extent to which drug formulations can be tested in young subjects, preventing a thorough characterization of medicines and excipients.

As a result, health care professionals often have had no recourse but to prescribe medicines to children off label, despite being unfamiliar with the true hazards of such formulations in children. An estimated 45% to 60% of drugs in the European Union are used off label in children, rising to 90% for neonates and infants [43]. This practice, although it has provided young patients with much-needed therapies, for whom treatments would otherwise be unavailable, is fraught with peril due to the initial lack of oversight and regulations that normally govern approved indicated uses for therapeutics.

Paediatric Regulation No 1901/2006 [44], by the European Commission, was borne from these concerns to establish guidelines for developing and authorizing safe, effective, and age-appropriate medicines for children. Based on this initiative, the EMA created its Pediatric Committee and mandated that a pediatric investigation plan (PIP) be filed to detail all preclinical and clinical activity, including formulation and excipients, concerning the development of drugs for pediatric use [45]. This measure followed an initiative by the US FDA, with its requirement of a pediatric study plan (PSP), as legislated by the Pediatric Research Equity Act [46]. Together, the PIP and PSP outline the demands of health authorities in ensuring that the necessary data are obtained in developing any drug that could benefit children.

However, as discussed by a recent multinational panel [47], assessing the safety of excipients in children is particularly challenging, primarily due to the lack of comprehensive safety data and standardized guidelines for acceptable risk-benefit profiles of pediatric excipient use. Ideally, safety data will exist in the literature and in databases, such as the Safety & Toxicity of Excipients for Pediatrics (STEP) database [48]. Recent initiatives [40], such as the Safe Excipient Exposure in Neonates and Small Children (SEEN) project [49], are compiling and quantifying excipient use among neonates and young pediatric patients to guide clinicians toward medications with the best risk-benefit profile, which will depend heavily on context, i.e., the condition that is being treated and the anticipated outcome.

For instance, concerning the pediatric population, for whom there are still frequently few approved, properly dosed medications, patient access becomes a critical issue, in that the options that are available to young recipients might have excipient levels that exceed the content that is known to be safe. But in the absence of alternatives, clinicians might have no choice but to administer them, particularly in the case of a life-threatening disease. With regard to PSs, the likelihood of their rare toxicity events must be weighed against their benefits.

Ultimately, the goal is to establish safe doses for excipients in pediatric formulations, one method for which is to refer to the maximum adult doses that are applied in marketed drug products, if only as an initial estimate, and, as discussed above, to identify specific thresholds from pediatric clinical data that demarcate the (sometimes age-specific) levels that elicit each degree in the spectrum of adverse effects (severe, less severe, long-term, short-term).

As suggested by a 2015 review [50], toxicity studies in juvenile animals might be warranted to generate nonclinical data when there are insufficient animal and human data to support the safe use of an excipient in pediatric formulations. In such cases, nonclinical data and human data on nonmedicinal exposure to excipients (such as from foods) can be applied, although the former may have limited value for PS, because anaphylaxis and hypersensitivity are difficult and often impossible to examine preclinically and are specific to the route of administration (parenteral vs. oral).

As in determining the maximum safe starting dose of a therapeutic in adult phase I clinical trials [51], one element in the extrapolation of these data is the use of safety (or uncertainty or adjustment) factors [52,53] that incorporate a margin of safety to account for various uncertainties in the extrapolation. Specifically, a margin of safety represents a buffer between the safe levels to which patients can be exposed and the amount of compound that has been documented to be associated with signs of toxicity, giving clinicians a certain degree of latitude and confidence that the risk of overexposing them to excipients will not be breached.

Ivanovska et al. have discussed the demand and urgency for pediatric drug formulations, citing several age-specific versions that are being developed [54]. However, the process of clinical trial testing, regulatory approval, and authorization is lengthy, and even medicines that are approved for pediatric use might be improperly dosed for certain age brackets, necessitating a faster, more straightforward approach to providing safe and age-appropriate doses of drug formulations to young patients.

### 3.4. The Progressive Pediatric Safety Factor

To this end, we have developed the progressive pediatric safety factor (PPSF) to aid formulation experts and clinicians in quickly estimating the potential maximum age-specific doses of excipients in the absence of reliable safety information for the pediatric patient population.

To define the maximum recommended starting dose (MRSD) for a clinical trial, an interspecies allometric scale was applied to convert doses from animal to human studies. Starting from the no-observed-adverse-effect level (NOAEL) of a drug from preclinical toxicological studies, a human equivalent dose (HED) is estimated. The HED is divided by a factor of 10 to increase the safety of the first human dose, accounting for differences in physiology and pharmacokinetics/pharmacodynamics between human and animal species, because the shift in species represents a stepwise change [55].

In the case of pediatric administration, active pharmaceutical ingredient (API) dosing is adapted. Whereas dosing that is based on body surface area might be preferred, clinical experience indicates that errors in measuring height, length (particularly in smaller children and infants), and body surface area from weight and height are common [56]. Thus, because API dosing is, in practice, based on body weight, the same can be done for excipients—not to ensure the same activity but the same innocuity as in adults. This simple adaptation to weight, however, is no longer sufficient, because differences in physiological and biological processes in the child are likely, compared with the adult. Nevertheless, these disparities will be less significant than when changing the species, as in the case of interspecies allometric scaling. Moreover, the change will not occur at a specific age or weight of the child but will become progressively significant the more immature an organ is, especially the liver and kidney—the primary organs of detoxification.

Neonates are assigned a PPSF of 10 (i.e., the full, generally applied uncertainty factor, widely used as default factor to cover interspecies differences, inter-individual variability or other uncertainties such as data robustness), reflecting completely immature detoxification systems, after which the PPSF decreases progressively by age to a value of 1 in subjects aged 2 years and older—the age at which detoxification processes have generally matured to adult levels (Table 1). As with other safety factors, the PPSF includes a margin of safety to allow for individual differences in maturation and development at specific ages—the PPSF does not merely follow the maturation curve for detoxification processes but lags slightly, essentially underestimating the actual maturation that has occurred and thus allowing the clinician to err on the side of caution. For example, at age 1.5 years, the PPSF is 2.3, accounting for those whose detoxification processes have not matured fully, despite the maturation curve reaching 100% by age 12 months for major detoxification systems.

This cutoff is based on the gestational and developmental growth of the kidney and liver in humans. With regard to kidney function, nephrogenesis is completed in utero, but functional maturity, in terms of glomerular filtration rate, reaches adult levels by age 3–5 months, and the ability to excrete concentrated urine and a sodium load is acquired by age 1 year [34,35,57]. Further, after approximately 6 months, the renal parenchyma in the infant kidney resembles that in adults [58], and tubular function has matured [35]. Conversely, the liver can take up to 2 years to reach full maturity [59]. By age 1–3 years, the activity of drug-metabolizing enzymes nears that in adults [35].

The advantage of the PPSF is that it can be applied generically for other excipients that are metabolized and excreted by the liver and kidney to determine their initial feasibility, because it is not based on compound-specific data and can refer to the available safety data of the excipient for adults. An assessment of their inherent safety, however, would need to be performed to determine the dose at which no safety factor would have to be applied, i.e., the maximum dose in adults that is well tolerated at a given route of administration. Further, the PPSF yields a binary response, independent of an excipient’s attributes, informing the user on whether it is likely to be safe (or not) at a specific dose at a certain age. Based on this applicability, the PPSF could be considered justification for the deliberation and use of an excipient in communicating with regulatory agencies, particularly before submission of a PSP or PIP.

Thus, the PPSF is a pragmatic approach that is well suited for easy use in the risk analysis stage of the assessment of excipients and perhaps by the health care professionals who dispense and administer medicines to neonates and infants.

### 3.5. Practical Considerations for the PPSF

For patients aged up to 2 years, the PPSF is used to determine the maximum acceptable excipient dose (MAED), calculated as follows:

(1) $$MAED=\left(\frac{MAED_{A}}{PPSF}\right)\times \left(\frac{BW_{S}}{BW_{A}}\right)$$

where MAED is the maximum acceptable excipient dose (MAEDA is that for adults) and BWS and BWA are the body weight of the subject and average adult, respectively.

**Figure 1:**
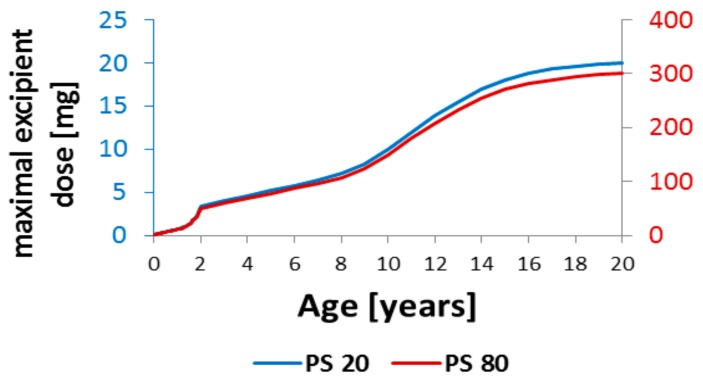
Maximum acceptable doses of parenterally administered Polysorbate 80 (PS80) and Polysorbate 20 (PS20) by age. Up to age 2 years, the maximum acceptable excipient dose (MAED) is based on the PPSF and body weight and solely on body weight thereafter.

Based on the known maximum tolerated doses of PS20 and PS80 for adults (300 mg and 20 mg, respectively), the maximum PS doses can be calculated for every increment in age up to 2 years; Table 2 lists the range of PPSF values and the resulting maximum acceptable doses of PS for neonates, infants, and toddlers, and Figure 1 conveniently charts these doses as a function of age.

Compared with the NOAEL of a retrospective analysis [60] and the limits that have been established by the Paediatric Committee (PDCO) of the EMA, the PPSF yields maximum PS doses that are far more conservative, implying that the PPSF embodies an additional safety factor that generates safer doses beyond what is already accepted. As shown in the example in Table 3, for a newborn (age 0 days), weighing 3.3 kg, using a PPSF of 10, the resulting maximum acceptable doses of PS80 and PS20 are 1.4 mg and 0.09 mg, respectively, compared with 9.90 mg per the PDCO—significantly less PS than what was coadministered in E-Ferol to neonates [61]. Thus, our model is highly conservative, essentially increasing the safety factor by 7–110 in this example (i.e., the ratio between the maximum doses as calculated by the PDCO and PPSF model), comprising additional safety margins that account for the immaturity of major detoxification systems in neonates and infants and the projections of existing models. Table 4 lists representative PPSF-derived doses of PS20 and PS80 for various ages.

To apply the PPSF to any other relevant excipient (except those that are not metabolized via the liver or kidney), one merely enters the maximum tolerated adult dose at a given route of administration for the compound of interest and the weight of the patient into the formula above.

## 4. Conclusions

The PPSF is intended to be used as an ancillary tool for those who are involved in the early phases of the development of formulations for pediatric patients, particularly with regard to generating pediatric plans for regulatory health authorities. Typically, proposals of formulations for pediatric use are submitted after the Phase I or II trial is completed, but in the absence of existing supportive safety data, they can invariably be rejected by health authorities. Consequently, the consideration of pediatric use of formulations may need to be supported by alternate pretexts.

The PPSF can serve as one such justification to bolster the rationale for pediatric formulations. Although it is not intended to replace or be used instead of compound-specific data and extrapolation models (e.g., PBPK), it can provide pharmaceutical developers and authorities with a quick and conservative preliminary assessment of whether it is feasible and likely to be safe to consider a pediatric formulation with particular levels of excipients. Specifically, the PPSF conservatively estimates safe levels of PSs for patients up to age 2 years, essentially generating a starting point for what are considered safe doses of PSs.

The limits on PS levels (or any other excipient) in currently available drugs that are established by the PPSF, however, do not imply that a formulation with higher amounts will be toxic, merely that such levels have not been tested.

## Figures and Tables

**Table 1:** Progressive pediatric safety factor (PPSF) values by age.

Age Category	Week	Year	Month	Height	Weight	PPSF
newborn infants (0 to 27 days)	0	0.00	0.0 months	49.5 cm	3.3 kg	10.0
	1	0.02	0.2 months	50.7 cm	3.4 kg	9.9
	2	0.04	0.5 months	51.9 cm	3.7 kg	9.6
	3	0.06	0.7 months	52.9 cm	3.9 kg	9.4
	4	0.08	0.9 months	53.9 cm	4.2 kg	9.0
infants and toddlers (28 days to 23 months)	5	0.10	1.2 months	54.8 cm	4.5 kg	8.7
	6	0.12	1.4 months	55.6 cm	4.8 kg	8.4
	7	0.13	1.6 months	56.5 cm	5.0 kg	8.2
	8	0.15	1.8 months	57.2 cm	5.2 kg	8.0
	9	0.17	2.1 months	58.0 cm	5.4 kg	7.8
	10	0.19	2.3 months	58.7 cm	5.6 kg	7.6
	11	0.21	2.5 months	59.3 cm	5.8 kg	7.4
	12	0.23	2.8 months	60.0 cm	5.9 kg	7.2
	13	0.25	3.0 months	60.6 cm	6.1 kg	7.0
		0.33	4 months	63.0 cm	6.7 kg	6.4
		0.42	5 months	65.0 cm	7.2 kg	5.9
		0.50	6 months	66.7 cm	7.6 kg	5.4
		0.58	7 months	68.2 cm	8.0 kg	5.0
		0.67	8 months	69.7 cm	8.3 kg	4.7
		0.75	9 months	71.1 cm	8.6 kg	4.4
		0.83	10 months	72.4 cm	8.8 kg	4.2
		0.92	11 months	73.7 cm	9.1 kg	3.9
		1.00	12 months	74.9 cm	9.3 kg	3.6
		1.08	13 months	76.1 cm	9.5 kg	3.4
		1.17	14 months	77.2 cm	9.7 kg	3.2
		1.25	15 months	78.3 cm	10.0 kg	2.9
		1.33	16 months	79.4 cm	10.2 kg	2.7
		1.42	17 months	80.5 cm	10.4 kg	2.5
		1.50	18 months	81.5 cm	10.6 kg	2.3
		1.58	19 months	82.5 cm	10.8 kg	2.1
		1.67	20 months	83.5 cm	11.0 kg	1.8
		1.75	21 months	84.4 cm	11.2 kg	1.6
		1.83	22 months	85.3 cm	11.4 kg	1.4
		1.92	23 months	86.2 cm	11.6 kg	1.2
children		2	24 months	86.4 cm	11.8 kg	1.0

**Table 2:** Maximum acceptable doses of PS20 (Polysorbate 20) and PS80 (Polysorbate 80), based on age and PPSF. Doses scaled down from 300 mg and 20 mg—the reference doses of PS80 and PS20 in amiodarone hydrochloride injection and doxercalciferol injection, respectively.

Age Category	Week	Year	PPSF	Polysorbate 20	Polysorbate 80
newborn infants (0 to 27 days)	0	0.00	10.0	0.09 mg	1.4 mg
	1	0.02	9.9	0.10 mg	1.5 mg
	2	0.04	9.6	0.11 mg	1.7 mg
	3	0.06	9.4	0.12 mg	1.8 mg
	4	0.08	9.0	0.13 mg	2.0 mg
infants and toddlers (28 days to 23 months)	5	0.10	8.7	0.15 mg	2.2 mg
	6	0.12	8.4	0.16 mg	2.4 mg
	7	0.13	8.2	0.17 mg	2.6 mg
	8	0.15	8.0	0.19 mg	2.8 mg
	9	0.17	7.8	0.20 mg	3.0 mg
	10	0.19	7.6	0.21 mg	3.2 mg
	11	0.21	7.4	0.22 mg	3.4 mg
	12	0.23	7.2	0.23 mg	3.5 mg
	13	0.25	7.0	0.25 mg	3.7 mg
		0.33	6.4	0.30 mg	4.5 mg
		0.42	5.9	0.35 mg	5.2 mg
		0.50	5.4	0.40 mg	6.0 mg
		0.58	5.0	0.46 mg	6.9 mg
		0.67	4.7	0.50 mg	7.6 mg
		0.75	4.4	0.56 mg	8.4 mg
		0.83	4.2	0.60 mg	9.0 mg
		0.92	3.9	0.67 mg	10.0 mg
		1.00	3.6	0.74 mg	11.1 mg
		1.08	3.4	0.80 mg	12.0 mg
		1.17	3.2	0.87 mg	13.0 mg
		1.25	2.9	0.99 mg	14.8 mg
		1.33	2.7	1.08 mg	16.2 mg
		1.42	2.5	1.19 mg	17.8 mg
		1.50	2.3	1.32 mg	19.8 mg
		1.58	2.1	1.47 mg	22.0 mg
		1.67	1.8	1.75 mg	26.2 mg
		1.75	1.6	2.00 mg	30.0 mg
		1.83	1.4	2.33 mg	34.9 mg
		1.92	1.2	2.76 mg	41.4 mg
children		2	1.0	3.37 mg	50.6 mg
(2–11 years)		3	1.0	4.03 mg	60.4 mg
		4	1.0	4.63 mg	69.4 mg
		5	1.0	5.23 mg	78.4 mg
		6	1.0	5.80 mg	87.0 mg
		7	1.0	6.46 mg	96.9 mg
		8	1.0	7.20 mg	108.0 mg
		9	1.0	8.29 mg	124.3 mg
		10	1.0	10.00 mg	150.0 mg
		11	1.0	12.00 mg	180.0 mg
adolescents		12	1.0	13.94 mg	209.1 mg
(12–18 years)		13	1.0	15.51 mg	232.7 mg
		14	1.0	17.00 mg	255.0 mg
		15	1.0	18.06 mg	270.9 mg
		16	1.0	18.83 mg	282.4 mg
		17	1.0	19.29 mg	289.3 mg
		18	1.0	19.57 mg	293.6 mg
adults		19	1.0	19.86 mg	297.9 mg
		20	1.0	20 mg	300 mg

**Table 3:** Estimated maximum acceptable daily doses of parenterally administered PS20 and PS80 by three models.

	PS20	PS80
NOAEL	8 mg	72 mg
PDCO	9.9 mg	9.9 mg
PPSF *	0.09 mg	1.4 mg

Abbreviations: NOAEL = no-observed-adverse-effect level, PDCO = Paediatric Committee of the European Medicines Agency * For a neonate, age 0 days, weighing 3.3 kg, using a Pediatric Progressive Safety Fact (PPSF) of 10.0.

**Table 4:** Maximum total daily doses of parenterally administered PS20 and PS80 at various ages, per the PPSF.

Age	PS20	PS80
28 days	0.13 mg	2.0 mg
6 months	0.4 mg	6.0 mg
12 months	0.74 mg	11.1 mg
24 months	3.37 mg	50.6 mg
